# Systematic Review and Meta Analysis of the Relative Effect on Plaque Index among Pediatric Patients Using Powered (Electric) versus Manual Toothbrushes

**DOI:** 10.3390/dj11020046

**Published:** 2023-02-09

**Authors:** Andrew Graves, Troy Grahl, Mark Keiserman, Karl Kingsley

**Affiliations:** 1 Department of Advanced Education in Pediatric Dentistry, School of Dental Medicine, University of Nevada-Las Vegas, 1700 W. Charleston Boulevard, Las Vegas, NV 89106, USA; 2 Department of Clinical Sciences, School of Dental Medicine, University of Nevada-Las Vegas, 1700 W. Charleston Boulevard, Las Vegas, NV 89106, USA; 3 Department of Biomedical Sciences, School of Dental Medicine, University of Nevada-Las Vegas, 1001 Shadow Lane, Las Vegas, NV 89106, USA

**Keywords:** systematic review, meta analysis, electric toothbrush, pediatric dental patient

## Abstract

Although many randomized controlled trials (RCT) have evaluated the efficacy of powered or electric toothbrushes compared with manual or traditional toothbrushes to remove biofilm and plaque, only one systematic review has been published for pediatric patients. The primary objective of this study was to perform a systematic review and meta analysis for this population. Using the PRISMA (Preferred Reporting Items for Systematic Reviews and Meta-Analyses) protocol, N = 321 studies were initially identified. Three independent, blinded abstract reviews were completed resulting in a total of n = 38/322 or 11.8% for the final analysis (n = 27 non-orthodontic, n = 11 orthodontic studies). Meta analysis of these outcome data have revealed a strong reduction in plaque index scores among pediatric patients using electric toothbrushes of approximately 17.2% for non-orthodontic patients and 13.9% for orthodontic patients. These results provide strong clinical evidence for recommending electric toothbrushing to pediatric patients, as well as those patients undergoing orthodontic therapy and treatment.

## 1. Introduction

Many randomized controlled trials (RCT) have evaluated the efficacy of powered or electric toothbrushes compared with manual or traditional toothbrushes to remove biofilm and plaque [1,2]. As many of these studies involved adults, there are also multiple lines of evidence to demonstrate the effect of powered or electric toothbrushes to improve or prevent gingivitis and early periodontitis [3,4,5]. The depth and breadth of research in this area has allowed multiple systematic reviews and meta analyses to be conducted on this topic, mainly focusing on adult patients and the improvements to oral health [6,7,8].

Due to the breadth and depth of research on the effectiveness of electric (powered) toothbrushes to reduce biofilm and plaque in adults, many research groups have also evaluated these effects among adolescents—although most of this research has focused on orthodontic patients [9,10,11]. These studies have also led to systematic reviews and meta analyses that provide for plaque control and biofilm reduction among this specific population, which includes both pediatric and adult patients [12,13,14]. However, the evidence comparing manual versus electric (powered) toothbrushes among pediatric patients without fixed orthodontic appliances is less robust [15,16,17].

However, despite evidence comparing these methods in pediatric patients, only one systematic review has been published for this specific population subgroup [18]. Although this review encompassed nine well-executed studies on the effectiveness of electric versus manual toothbrushing in pediatric patients, an expanded search has revealed additional studies published before and after this review was completed, which significantly increase the total number of available studies for inclusion [15,16,17]. In addition, an analysis of the cited references from each of these studies also led to additional resources for this systematic review. Finally, the evidence of strong correlation between the major plaque indices, including the Rustogi et al. Modified Navy Plaque Index (RMNPI) and the Turesky et al. Modification of the Quigley Hein Plaque Index(TQHPI), allowed for these many differing studies to be analyzed in this review [19]. 

Based upon this information, the primary objective of this study is to perform an expanded systematic review and meta analysis to answer the clinical research question “Do pediatric patients (Population) using powered or electric toothbrushes (Intervention) compared with manual or traditional toothbrushes (Comparison) exhibit reduced plaque indices (Outcome).”

## 2. Materials and Methods

### 2.1. Human Subjects Approval

This study was reviewed and approved as Exempt by the Institutional Review Board (IRB) and Office for the Protection of Research Subjects (OPRS) under protocol [1619329-1] titled Retrospective analysis of Oral Health Status of Dental Population on 24 July 2020. 

### 2.2. PRISMA Protocol

This study followed the Preferred Reporting Items for Systematic Reviews and Meta-Analysis (PRISMA) guidelines and protocol [20,21]. The National Library of Medicine (NLM) and PubMed databases were searched using Medical Subject Headings (MeSH), which are appropriate for controlled vocabulary indexing of published evidence relating to pediatric toothbrushing. The Boolean search terms used to search for relevant articles included the following, using AND/OR operators: “Pediatric”, “Children”, “Electric toothbrush”, “Powered toothbrush”, “Manual toothbrush”, “Traditional toothbrush”, “Primary dentition”, and “Mixed dentition”. Cited references from each of the studies identified were also evaluated to determine any potential resources not identified through the initial database search results.

The inclusion criteria were those related to human subjects that were pediatric patients (<18 years of age) in a dental setting using manual versus electric toothbrushes. Exclusion criteria included non-human subjects (animal or in vitro studies), studies involving only adult patients (>18 years of age), non-dental clinical applications (ex vivo), or study settings involving a second party, such as disabled or physically impaired patients. Studies that did not include measurements of plaque index, including Rustogi et al. Modified Navy Plaque Index (RMNPI), Silness-Loe plaque index (SLPI), or the Turesky et al. Modification of the Quigley Hein Plaque Index (TQHPI), were also excluded. All relevant articles were imported into an online system (Rayyan.ai) for comparison and analysis by the study authors.

### 2.3. Two-Step Review

This process was initially performed by the lead author and then separately and independently performed and confirmed by the second and third authors. Any discrepancies and questions were marked for discussion and review, which did not influence the final articles selected. The final articles selected were agreed upon by all three reviewers prior to the full-text review of each study.

### 2.4. Data Analysis

Data regarding plaque index were extracted from each study and presented in both table and graphic formats. Baseline (starting) measurements were compared with manual (control) and electric or powered (experimental) toothbrushing for each study endpoint (outcome). To enable analysis of studies using different plaque indices, including the Rustogi et al. Modified Navy Plaque Index or RMNPI, Silness-Loe plaque index or SLPI, and the Turesky et al. modification of the Quigley Hein Plaque Index or TQHPI, percent changes between baseline and control or experimental groups were used to determine the relative effect (RE) of electric versus manual toothbrush use. Pediatric studies including patients undergoing orthodontic therapy that involved the use of fixed brackets were analyzed separately from the studies involving pediatric patients without any orthodontic appliances.

## 3. Results

**Figure 1:**
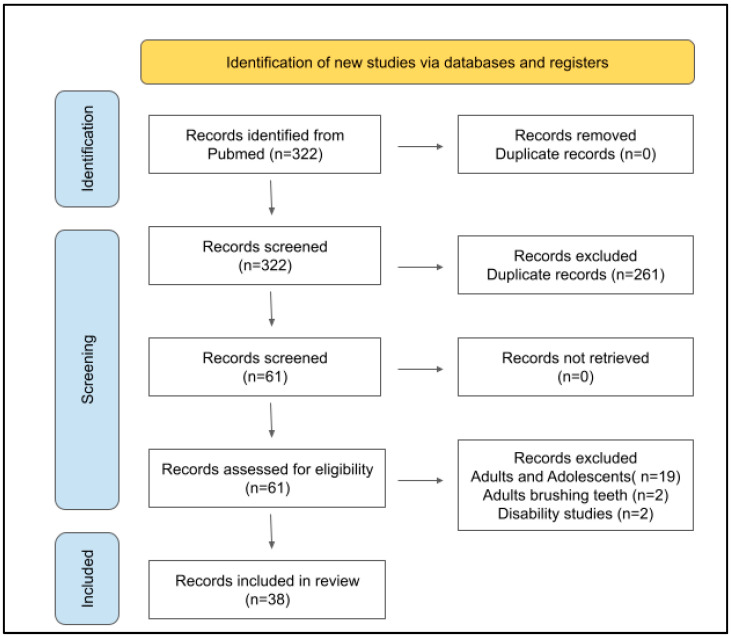
PRISMA protocol flow diagram of the literature review. Using the Preferred Reporting Items for Systematic Reviews and Meta-Analyses (PRISMA) protocol, the search in PubMed yielded a total of n = 322 research papers. Application of the inclusion and exclusion criteria reduced the number of articles to n = 60 for full-text retrieval. Final review of these studies and outcome measures resulted in n = 38/322 or 11.8% included in this systematic review.

Using the PRISMA protocol and PubMed search strategy, a total of n = 309 citations were found using the operators “Pediatric”, “Electric toothbrush”, “Powered toothbrush”, “Manual toothbrush”, and “Traditional toothbrush” and no duplicate entries were identified (Figure 1). The results were then screened by each reviewer independently, which resulted in more than half of the citations being excluded by all three reviewers upon initial review (n = 194/322 or 60.2%). The remaining studies where one or two reviewers disagreed (n = 67/322 or 20.8%) were reviewed together by all three reviewers. The total number of studies excluded at the review stage was n = 261/322 or 81.1%, with n = 61/322 or 18.9% marked for further review and full-text analysis.

Following the retrieval of full-text articles for all studies, n = 19 studies were excluded after review of the study parameters including many studies involving participants that included both adolescents and adults over 18 years of age. In addition, two studies were excluded from this review following data extraction, which revealed these studies included adults brushing the teeth for their children and two more studies involved children that were disabled and unable to brush their teeth without assistance. The remaining studies n = 38/322 or 11.8% were included in this review.

Full text reviews of all non-orthodontic pediatric studies (n = 27) were completed (Table 1). The analysis of data from all studies provided the year of the study publication, which ranged from 1967 to 2021. Sample sizes from each study were also evaluated, which ranged from n = 12 to n = 200 and averaged n = 60.2. The age ranges from each study varied from patients as young as two years old up to and including patients 17 years of age. Finally, all studies included in this systematic review had baseline and final outcome measures for control (manual) and experimental (electric/powered) groups using versions of the Rustogi et al. Modified Navy Plaque Index (RMNPI), the Silness-Loe plaque index (SLPI), or the Turesky et al. Modification of the Quigley Hein Plaque Index (TQHPI) to provide systematic objective criteria for comparison. These data demonstrated relative effects (electric versus manual toothbrush) on plaque index outcomes ranging from 0.5% to 35.4% that averaged 17.2%.

**Figure 2:**
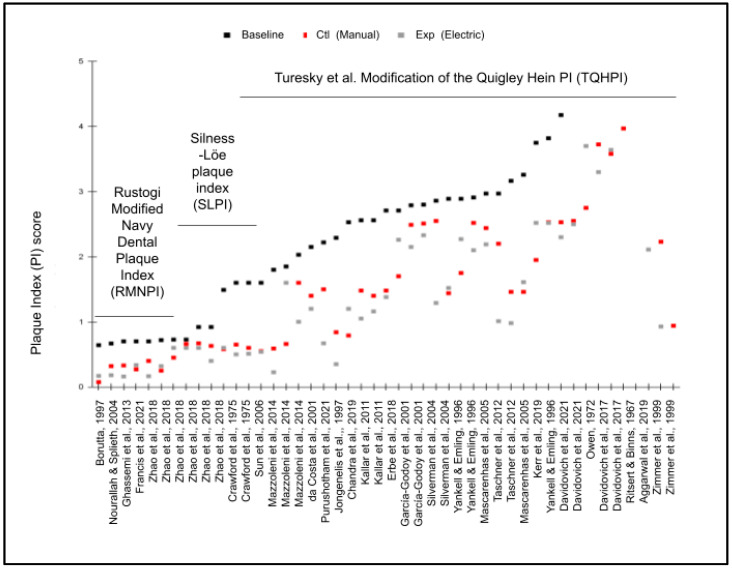
Graphic display of baseline and endpoint plaque indices. Baseline and endpoints from studies with Rustogi et al. Modified Navy Plaque Index (RMNPI, n = 8), Silness-Loe plaque index (SLPI, n = 6), and the Turesky et al. Modification of the Quigley Hein Plaque Index (TQHPI, n = 30) each demonstrated more significant reductions in the plaque index with the experimental variable (Exp) electric toothbrushes compared with the control (Ctl) variable manual toothbrushes [22,23,24,25,26,27,29,30,31,32,33,34,35,36,37,38,39,40,41,42,43,44,45,46,47,48].

The plaque index data were extracted from each of the studies where baseline and endpoint plaque indices were provided (Figure 2). Four of the studies used the Rustogi et al. Modified Navy Plaque Index (RMNPI), four used the Silness-Loe plaque index (SLPI), with most of the remainder (n = 15) using the Turesky et al. Modification of the Quigley Hein Plaque Index (TQHPI). Although some of the studies included multiple outcome data points (different trial lengths) and scales differed among each of the individual studies, these results demonstrate that every study demonstrated more significant reductions in the plaque index using electric toothbrushes compared with manual toothbrushes.

**Figure 3:**
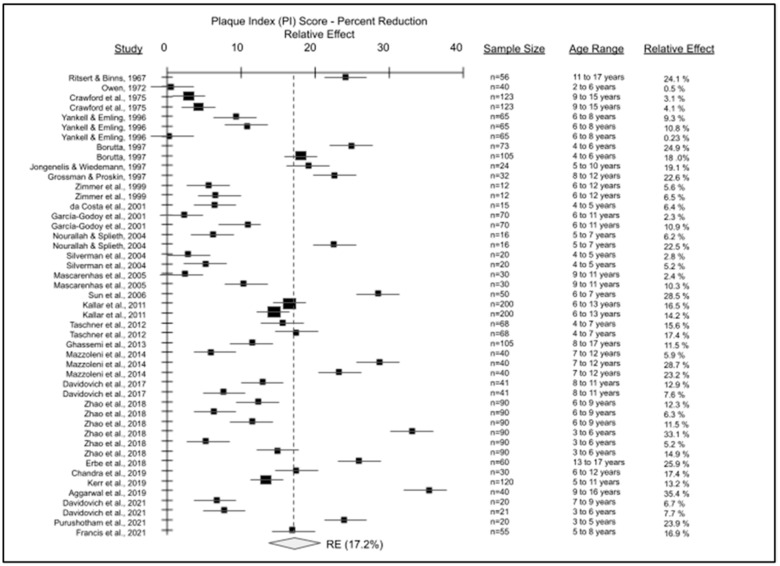
Forest plot of pediatric, non-orthodontic studies of manual versus electric toothbrushing comparing plaque index (PI). A total of 27 studies were evaluated with sample sizes ranging from n = 12 to n = 200 were plotted to determine an average reduction in plaque index or relative effect (RE) with electric toothbrush use of approximately 17.2%, *p* = 0.0073 [22,23,24,25,26,27,28,29,30,31,32,33,34,35,36,37,38,39,40,41,42,43,44,45,46,47,48].

The data from the final articles included for pediatric, non-orthodontic studies were combined to create the forest plot of plaque index score reductions comparing electric versus manual toothbrushes (Figure 3). Detailed analysis of the forest plot for the meta analysis outcome variable has revealed a strong reduction in plaque index scores among pediatric patients using electric toothbrushes compared with manual toothbrushes. Comparison of the baseline measurements compared with the manual toothbrush endpoint measurements revealed no statistical significance (*p* = 0.065), but comparison of baseline measurements with electric toothbrush endpoint measurements revealed statistically significant results (*p* = 0.0073). Percent reduction in plaque index scores from these RCT demonstrated comparative reductions in plaque with electric versus manual toothbrushes ranging from 0.23% to 35.4%, yielding an average reduction or relative effect (RE) of 17.2%. 

**Figure 4:**
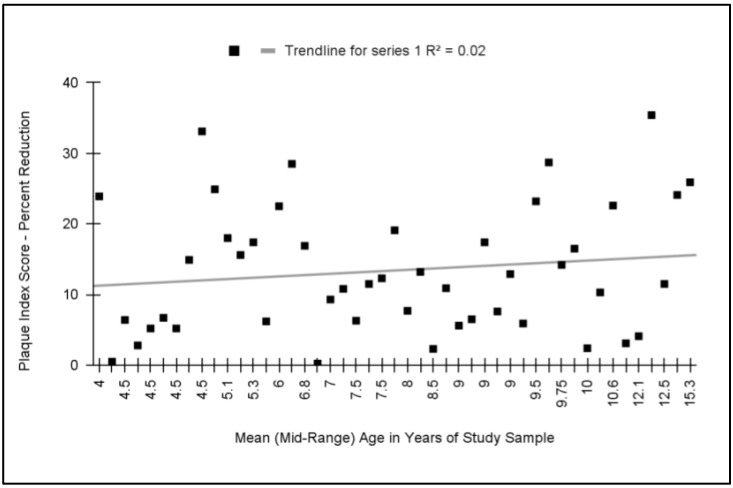
Meta analysis of relative effect (percent reduction in plaque index) plotted against average or mid-range age of study participants. No significant association was found between these variables with a coefficient of determination, R^2^ = 0.02. Studies with the average age of participants under six and over six years of age exhibited similar reductions in plaque index with electric toothbrushes (13.5% and 13.3%, respectively), *p* = 0.899.

To determine if any relationship exists between the relative effect and the mean or mid-range age of the study sample participants, these data were graphed and analyzed (Figure 4). The analysis of these data demonstrated that no significant relationship exists between these variables with a coefficient of determination (R^2^ = 0.02). Outcomes from studies with the average or mid-range age of participants between the ages of two and six (n = 16) exhibited an average relative reduction in plaque index (comparing electric with manual toothbrushes) of 13.6%, which was not significantly different from those outcomes from studies with the average or mid-range age of participants between six and seventeen years old (n = 30) that exhibited a reduction of 13.3%, *p* = 0.899. 

**Figure 5:**
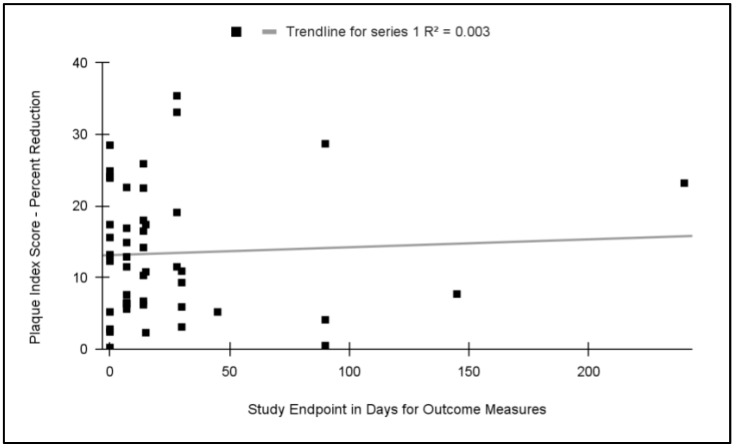
Meta analysis of relative effect (percent reduction in plaque index) plotted against study length (endpoint measures). No significant association was found between these variables with a coefficient of determination, R^2^ = 0.003. Studies lasting 0 days exhibited similar average plaque reduction effects (14.2%) as those lasting 7 to 14 days (13.8%), *p* = 0.711, or 30 days and longer (14.1%), *p* = 0.92.

To evaluate the potential effects on the study outcomes related to the length of study (endpoint measures), these data were graphed and analyzed (Figure 5). This analysis revealed that no significant relationship exists between the length of the study and the relative effect (R^2^ = 0.003). More specifically, the average relative effect (plaque index reduction) among the 0 day trials (14.2%) was not significantly different from the average of trials lasting one to two weeks (13.8%), *p* = 0.711, or trials lasting 30 days or longer (14.1%), *p* = 0.92. 

**Figure 6:**
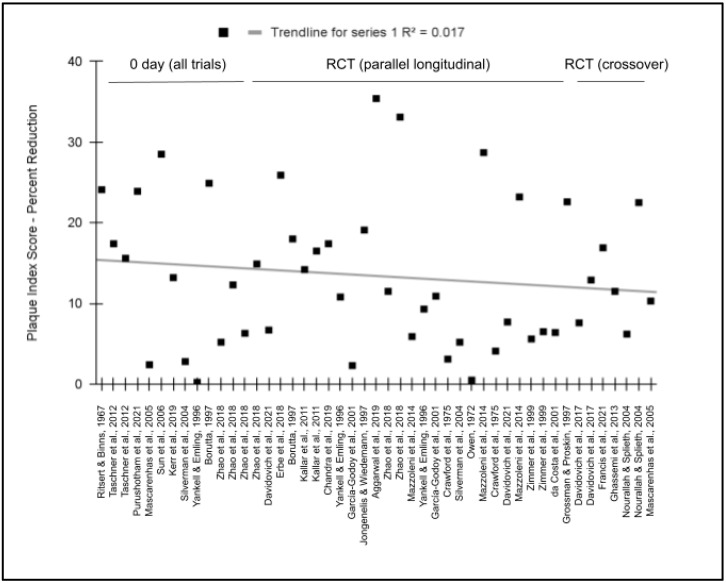
Meta analysis of relative effect (percent reduction in plaque index) plotted against study design. No significant association was found between these variables with a coefficient of determination, R^2^ = −0.017. Studies lasting 0 days exhibited similar average plaque reduction effects (14.2%) as longitudinal RCTs (13.2%), *p* = 0.767, and crossover RCTs (12.9%), *p* = 0.735 [22,23,24,25,26,27,28,29,30,31,32,33,34,35,36,37,38,39,40,41,42,43,44,45,46,47,48].

To evaluate the potential effects on the study outcomes related to the study design, these data were graphed and analyzed (Figure 6). This analysis revealed that no significant relationship exists between the type of the study (such as longitudinal parallel RCTs and crossover RCT) and the relative effect (R^2^ = −0.017). More specifically, the average relative effect (plaque index reduction) among all of the zero day trials (14.2%) was not significantly different from the average of longitudinal parallel RCTs lasting any length of time (13.2%), *p* = 0.767, or crossover RCTs regardless of length (12..9%), *p* = 0.735. 

Full text reviews of all orthodontic pediatric studies (n = 11) were completed (Table 2). Analysis of these studies revealed a range of publication dates between 1996 and 2021. Sample sizes from each study were also evaluated, which ranged from n = 20 to n = 80, averaging n = 45.5, and yielding a total combined sample size of n = 500. Finally, the comparison of control (manual) and experimental (electric/powered) groups using various plaque indexes, including the Silness-Loe plaque index (SLPI) and the Turesky et al. Modification of the Quigley Hein Plaque Index (TQHPI), demonstrated relative effects on plaque index scores for orthodontic patients ranging from −17.5% to 68.2% that averaged 13.9%.

**Figure 7:**
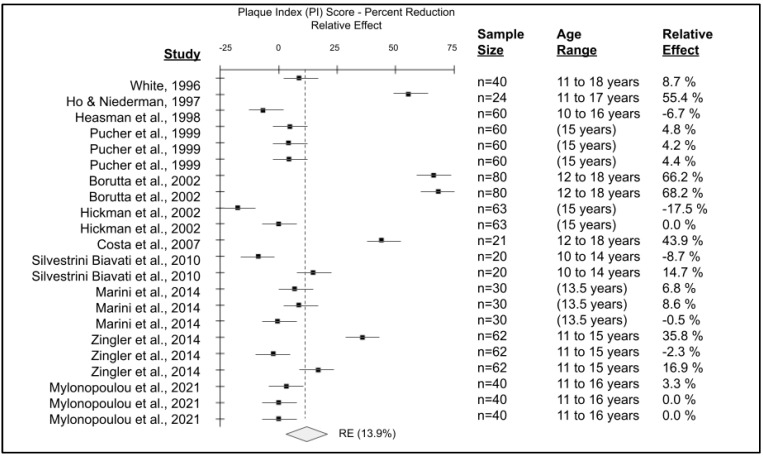
Forest plot of pediatric, orthodontic studies of manual versus electric toothbrushing comparing plaque index (PI). A total of n = 11 studies were evaluated with sample sizes ranging from n = 20 to n = 80 were plotted to determine an average reduction in plaque index or relative effect (RE) with electric toothbrush use of approximately 13.9%, *p* = 0.035 [49,50,51,52,53,54,55,56,57,58,59].

The data from the final articles included for pediatric, orthodontic studies were combined to create the forest plot of plaque index score reductions comparing electric versus manual toothbrushes (Figure 7). Detailed analysis of the outcome variable has revealed variable reductions in plaque index scores among pediatric orthodontic patients using electric toothbrushes compared with manual toothbrushes. Comparison of the baseline measurements compared with the manual toothbrush orthodontic endpoint measurements revealed no statistical significance (*p* = 0.12), but comparison of baseline measurements with electric toothbrush orthodontic endpoint measurements revealed statistically significant results (*p* = 0.035). Changes in plaque index scores from these RCT demonstrated comparative reductions in plaque with electric versus manual toothbrushes ranging from −17.5% to 68.2%, yielding an average reduction or relative effect (RE) of 13.9%.

Most of the studies had patients with similar ages, with the youngest patients in each RCT between 10 and 16 years of age and the oldest between 12 and 17.9 years of age. Analysis of study sample age and plaque index outcomes revealed no significant associations (R^2^ = 0.043). Similarly, analysis of sample size with plaque index outcomes revealed no significant association (R^2^ = 0.029), as most of the sample sizes were relatively small (between n = 20 and n = 80).

To evaluate the potential bias in all of the studies (non-orthodontic, orthodontic), specific characteristics of each study were extracted for comparison (Table 3). These data demonstrated that the majority of studies (n = 25/38 or 65.7%) included in this review had low selection bias (randomized controlled trials), although some studies did use randomized selection from a convenience sample (n = 12/38 or 34.2%). In addition, the vast majority (n = 36/38 or 94.7%) had low or very low attrition rates, which provides evidence that completion bias was not a significant influence on the results of these studies. Finally, most studies used two or more blinded operators for the evaluation of patients, with some studies using the same blinded operator (not knowing which group the patient belonged to).

## 4. Discussion

To date, few studies have systematically reviewed all relevant evidence to evaluate the clinical question regarding the relative effect of electric versus manual toothbrushes to reduce plaque indices within the pediatric patient population [18]. This review significantly increases the total number of subjects evaluated by nearly 1000 [22,23,24,28,32,35,36,37,38,39,40,41,42,43,44,45,46,47,48,49,50,51,52,53,54,55,56,57,58,59]. In addition, by focusing on this specific outcome measure (plaque index scores) the current systematic review also provides a more direct and meaningful comparison of clinical patient outcomes—an estimate of the average plaque coverage of all tooth surfaces expressed as either a numeric percentage or on a predefined scale (e.g., 0 to 3 or 0 to 5) [60,61].

These results suggest that even among children as young as two years of age, use of electric toothbrushes can provide significant improvements in plaque index score [6,18]. Moreover, these data suggest that these improvements appear to remain constant over the age range evaluated (2 to 17 years of age), which suggests that even very young patients may experience clinical improvements in plaque reduction from early introduction to powered toothbrushes [12,18]. In addition, the separate analysis of study length also suggests that clinically relevant reductions in plaque can be demonstrated as early as the first day, which continue regardless of the time period evaluated [60,61,62,63]

The results of this study also provided subgroup analysis of orthodontic pediatric patients from randomized controlled trials that also demonstrated clinical reduction in plaque index scores among non-orthodontic patients. These data confirm previous observations from systematic reviews and meta analyses, although the most recent of these focusing exclusively on pediatric patients was completed in 2008 [62,63]. Moreover, these results provide further support for the most recent published guidelines and recommendations from clinical practitioners regarding the recommendation for electric or powered toothbrushes for pediatric patients [13,64,65].

Despite the significance of these findings and the relevance of having an updated and expanded systematic review and meta analysis of these outcomes, there are some limitations implicit in this study that should also be considered. For example, many of these studies used different types of toothbrushes that may have influenced the specific outcomes in those RCT [66,67]. In addition, some of these studies also included hygiene instruction and other behavioral interventions that may have the potential to impact the outcomes measured in these trials [68,69]. Finally, differences in the study design, such as the number of clinical observers or operators, as well as minor differences in the type of plaque indices used, such as the percentage-based Simplified Oral Health Index or the scale-based indices including the Rustogi Modified Navy Plaque Index (RMNPI), Silness-Loe plaque index (SLPI), and the original or Turesky modification of the Quigley Hein Plaque Index (TQHPI) may produce small variations in outcome measurements [70].

## 5. Conclusions

This systematic review combines the results of multiple non-orthodontic and orthodontic studies to provide an updated and more expansive evaluation of the relative effectiveness of electric versus manual toothbrushes among pediatric patients. This analysis demonstrates the clinical utility of using electric toothbrushes among patients as young as two years of age—with strong evidence that these effects may be consistent up to age seventeen.

## Figures and Tables

**Table 1:** Characteristics of pediatric, non-orthodontic inclusion studies.

Study	Study Design	Sample Size	Age Range (Median)	Study Endpoint(s) Follow-Up Period(s)	Plaque Index Score—Percent Reduction
Ritsert and Binns, 1967 [22]	Split-mouth parallel single day trial	n = 56	11–17 years (13.5 years)	0 days	Silness and Loe (SLPI) (0 days) 24.1%
Owen, 1972 [23]	Randomized RCT parallel longitudinal trial	n = 40	2–6 years (4.3 years)	90 days	Modified Ramfjord (MRPI) (90 days) 0.5%
Crawford et al., 1975 [24]	Randomized RCT parallel longitudinal trial	n = 123	9–15 years (12.1 years)	30 days 90 days	Silness and Loe (SLPI) (30 days) 3.1% (90 days) 4.1%
Yankell and Emling, 1996 [25]	Randomized RCT parallel longitudinal trial	n = 65	6–8 years (7.0 years)	0 days 15 days 30 days	Turesky Modified Quigley Hein Plaque Index (TQHPI) (0 days) 0.23% (15 days) 10.8% (30 days) 9.3%
Borutta, 1997 [26]	Randomized RCT parallel longitudinal trial	n = 73	4–6 years (5.1 years)	0 days 14 days	Rustogi Modified Navy Plaque Index (RMNPI) (0 days) 24.9% (14 days) 18.0%
Jongenelis and Wiedemann, 1997 [27]	Randomized RCT parallel longitudinal trial	n = 24	5–10 years (7.8 years)	28 days	Turesky Modified Quigley Hein Plaque Index (TQHPI) (28 days) 19.1%
Grossman and Proskin, 1997 [28]	Randomized Crossover longitudinal trial	n = 32	8–12 years (10.6 years)	7 days	Global Plaque Index (GPI)(7 days) 22.6%
Zimmer et al., 1999 [29]	Randomized Crossover longitudinal trial	n = 12	6–12 years (9 years)	7 days	Turesky Modified Quigley Hein Plaque Index (TQHPI) Primary (7 days) 5.6% Mixed (7 days) 6.5%
da Costa et al., 2001 [30]	Randomized Crossover longitudinal trial	n = 15	4–5 years (4.5 years)	7 days	Silness and Loe (SLPI) (7 days) 6.4%
García-Godoy et al., 2001 [31]	Randomized RCT parallel longitudinal trial	n = 70	6–11 years (8.5 years)	15 days 30 days	Turesky Modified Quigley Hein Plaque Index (TQHPI) (15 days) 2.3% (30 days) 10.9%
Nourallah and Splieth, 2004 [32]	Randomized Crossover longitudinal trial	n = 16	5–7 years (6 years)	14 days	Occlusal Plaque Index (OPI) (14 days) 6.2% Approximal Plaque Index (API) (14 days) 22.5%
Silverman et al., 2004 [33]	Randomized RCT parallel longitudinal trial	n = 20	4–5 years (4.5 years)	0 days 45 days	Turesky Modified Quigley Hein Plaque Index (TQHPI) (0 days) 2.75% (45 days) 5.2%
Mascarenhas et al., 2005 [34]	Randomized Crossover longitudinal trial	n = 30	9–11 years (10 years)	0 days 14 days	Turesky Modified Quigley Hein Plaque Index (TQHPI) (0 days) 2.4% (14 days) 10.3%
Sun et al., 2006 [35]	Randomized RCT parallel single day trial	n = 50	6–7 years (6.5 years)	0 days	Turesky Modified Quigley Hein Plaque Index (TQHPI) (0 days) 28.5%
Kallar et al., 2011 [36]	Randomized RCT parallel longitudinal trial	n = 200	6–13 years (9.75 years)	14 days	Turesky Modified Quigley Hein Plaque Index (TQHPI) Supervised (14 days) 16.5% Unsupervised (14 days) 14.2%
Taschner et al., 2012 [37]	Split-mouth parallel single day trial	n = 68	4–7 years (5.3 years)	0 days	Turesky Modified Quigley Hein Plaque Index (TQHPI) (0 days; low speed) 15.6% (0 days; high speed) 17.4%
Ghassemi et al., 2013 [38]	Randomized Crossover longitudinal trial	n = 105	8–17 years (12.5 years)	7 days	Rustogi Modified Navy Plaque Index (RMNPI) (7 days) 11.5%
Mazzoleni et al., 2014 [39]	Randomized RCT parallel longitudinal trial	n = 40	7–12 years (9.5 years)	30 days 90 days 240 days	Silness and Loe (SLPI) (30 days) 5.9% (90 days) 28.7% (240 days) 23.2%
Davidovich et al., 2017 [40]	Randomized Crossover longitudinal trial	n = 41	8–11 years (9 years)	7 days	Turesky Modified Quigley Hein Plaque Index (TQHPI) (7 days; Mixed dentition) 12.9% (7 days; Permanent dentition) 7.6%
Zhao et al., 2018 [41]	Randomized RCT parallel longitudinal trial	n = 90 n = 90	3–6 years (4.5 years) 6–9 years (7.5 years)	0 days 7 days 28 days	Rustogi Modified Navy Plaque Index (RMNPI) 3–6 years (0 days) 5.2% (7 days) 14.9% (28 days) 33.1% 6–9 years (0 days) 12.3% (7 days) 6.3% (28 days) 11.5%
Erbe et al., 2018 [42]	Randomized RCT parallel longitudinal trial	n = 60	13–17 years (15.3 years)	14 days	Turesky Modified Quigley Hein Plaque Index (TQHPI) (14 days) 25.9%
Chandra et al., 2019 [43]	Randomized RCT parallel longitudinal trial	n = 30	6–12 years (9 years)	15 days	Turesky Modified Quigley Hein Plaque Index (TQHPI) (15 days) 17.4%
Kerr et al., 2019 [44]	Randomized RCT parallel single day trial	n = 120	5–11 years (8 years)	0 days	Simplified Oral Health Index (SOHI) (0 days) 13.2%
Aggarwal et al., 2019 [45]	Randomized RCT parallel longitudinal trial	n = 40	9–16 years (12.5 years)	28 days	Turesky Modified Quigley Hein Plaque Index (TQHPI) (28 days) 35.4%
Davidovich et al., 2021 [46]	Randomized RCT parallel longitudinal trial	n = 20 n = 21	3–6 years (4.5 years) 7–9 years (8 years)	7–14 days	Turesky Modified Quigley Hein Plaque Index (TQHPI) (14 days; 3–6 years) 6.7% (14 days; 7–9 years) 7.7%
Purushotham et al., 2021 [47]	RCT parallel single day trial	n = 20	3–5 years (4 years)	0 days	Turesky Modified Quigley Hein Plaque Index (TQHPI) (0 days) 23.9%
Francis et al., 2021 [48]	Randomized Crossover longitudinal trial	n = 55	5–8 years (6.8 years)	7 days	Rustogi Modified Navy Plaque Index (RMNPI) (7 days) 16.9%
**Total number of pediatric non-orthodontic studies n = 27** **(1967–2021)**		**Total: 1626** **Average: 60.2** **Range: ** **12–200**	**Average: ** **7.9 years** **Range: ** **2–17 years**	**Average:** **25.2 days** **Range:** **0–240 days**	**Average PI reduction: 17.2%**

**Table 2:** Characteristics of pediatric, orthodontic inclusion studies.

Study	Study Design	Sample Size	Age Range (Median)	Study Endpoint(s) Follow-up Period(s)	Plaque Index Score—Percent Reduction
White, 1996 [49]	Randomized RCT parallel longitudinal trial	n = 40	11–18 years (14.5)	84 days	Hygiene Analysis Index (HAI) 8.7%
Ho and Niederman, 1997 [50]	Randomized RCT parallel longitudinal trial	n = 24	11–17 years (14 years)	28 days	Silness and Loe (SLPI) 55.4%
Heasman et al., 1998 [51]	Randomized Crossover longitudinal trial	n = 60	10–16 years (13.6 years)	28 days	Visual Plaque Index (VPI) −6.7%
Pucher et al., 1999 [52]	Randomized RCT parallel longitudinal trial	n = 60	(15 years)	0 days 15 days 42 days	Turesky Modified Quigley Hein Plaque Index (TQHPI) (0 days) 4.8% (15 days) 4.2% (42 days) 4.4%
Borutta et al., 2002 [53]	Randomized RCT parallel longitudinal trial	n = 80	12–18 years (13.5 years)	14 days 28 days	Turesky Modified Quigley Hein Plaque Index (TQHPI) (14 days) 66.2% (28 days) 68.2%
Hickman et al., 2002 [54]	Randomized RCT parallel longitudinal trial	n = 63	(15 years)	28 days 56 days	Silness & Loe (SLPI) (28 days) −17.5% (28 days) 0.0%
Costa et al., 2007 [55]	Randomized RCT parallel longitudinal trial	n = 21	12–18 years (15.2 years)	30 days	Silness and Loe (SLPI) 43.9%
Silvestrini Biavati et al., 2010 [56]	Randomized RCT parallel longitudinal trial	n = 20	10–14 years (11.4 years)	14 days 28 days	O’Leary Plaque Index (OLPI) −8.7% 14.7%
Marini et al., 2014 [57]	Randomized RCT parallel longitudinal trial	n = 30	(13.5 years)	28 days 56 days 84 days	Turesky Modified Quigley Hein Plaque Index (TQHPI) (28 days) 6.8% (56 days) 8.6% (84 days) −0.5%
Zingler et al., 2014 [58]	Randomized RCT parallel longitudinal trial	n = 62	11–15 years (13.1 years)	28 days 56 days 84 days	Turesky Modified Quigley Hein Plaque Index (TQHPI) (28 days) 35.8% (56 days) 2.3% (84 days) 16.9%
Mylonopoulou et al., 2021 [59]	Randomized RCT parallel longitudinal trial	n = 40	11–16 years (14 years)	30 days 60 days 90 days	Silness & Loe (SLPI) (30 days) 3.3% (60 days) 0.0% (90 days) 0.0%
**Total number of orthodontic pediatric studies, n = 11** **(1996–2021)**		**Total: 500** **Average: 45.5** **Range: ** **20–80**	**Average: ** **13.9 years** **Range: ** **10–17 years**	**Average:** **61 days** **Range:** **0–84 days**	**Average PI reduction: 13.9%**

**Table 3:** Potential bias of all pediatric inclusion studies.

Study Authors	Selection Bias	Attrition Bias	Reporting (Observer) Bias
Ritsert and Binns, 1967 [22]	High (Randomized from Convenience sample)	Very low (Attrition 0%)	Unknown (Not reported)
Owen, 1972 [23]	High (Randomized from Convenience sample)	Very low (Attrition 5.0%)	Unknown (Not reported)
Crawford et al., 1975 [24]	Low (RCT)	Very low (Attrition 2.2%)	Low (Two independent operators)
Yankell and Emling, 1996 [25]	High (Randomized from Convenience sample)	Very low (Attrition 1.6%)	Low (Multiple operators)
Borutta, 1997 [26]	Low (RCT)	Low (Attrition 13.3%)	Low (Two independent operators)
Jongenelis and Wiedemann, 1997 [27]	High (Randomized from Convenience sample)	Very low (Attrition 1.6%)	Low (Multiple operators)
Grossman and Proskin, 1997 [28]	Low (RCT)	Very low (Attrition 0.0%)	Moderate (One blinded operator)
Zimmer et al., 1999 [29]	High (Randomized from Convenience sample)	Very low (Attrition 0.0%)	Moderate (One blinded operator)
da Costa et al., 2001 [30]	High (Randomized from Convenience sample)	Very low (Attrition 0.0%)	Moderate (One blinded operator)
García-Godoy et al., 2001 [31]	Low (RCT)	Very low (Attrition 5.3%)	Low (Multiple operators)
Nourallah and Splieth, 2004 [32]	High (Randomized from Convenience sample)	Very low (Attrition 0.0%)	Low (Multiple operators)
Silverman et al., 2004 [33]	High (Randomized from Convenience sample)	Very low (Attrition 3.4%)	Low (Multiple operators)
Mascarenhas et al., 2005 [34]	High (Randomized from Convenience sample)	Very low (Attrition 0.0%)	Moderate (One blinded operator)
Sun et al., 2006 [35]	High (Randomized from Convenience sample)	Very low (Attrition 0.0%)	Low (Multiple operators)
Kallar et al., 2011 [36]	Unknown	Very low (Attrition 0.0%)	Unknown (Not reported)
Taschner et al., 2012 [37]	Low (RCT)	Very low (Attrition 1.4%)	Moderate (One blinded operator)
Ghassemi et al., 2013 [38]	Low (RCT)	Very low (Attrition 1.9%)	Moderate (One blinded operator)
Mazzoleni et al., 2014 [39]	High (Randomized from Convenience sample)	Very low (Attrition 0.0%)	Moderate (One blinded operator)
Davidovich et al., 2017 [40]	Low (RCT)	Very low (Attrition 0.0%)	Low (Multiple operators)
Zhao et al., 2018 [41]	Unknown	Very low (Attrition 4.4%)	Unknown (Not reported)
Erbe et al., 2018 [42]	Low (RCT)	Very low (Attrition 1.7%)	Moderate (One blinded operator)
Chandra et al., 2019 [43]	Low (RCT)	Very low (Attrition 0.0%)	Unknown (Not reported)
Kerr et al., 2019 [44]	High (Randomized from Convenience sample)	Very low (Attrition 0.0%)	Low (Multiple operators)
Aggarwal et al., 2019 [45]	Low (RCT)	Very low (Attrition 0.0%)	Moderate (One blinded operator)
Davidovich et al., 2021 [46]	Low (RCT)	Low (Attrition 4.8%)	Moderate (One blinded operator)
Purushotham et al., 2021 [47]	Low (RCT)	Low (Attrition 5%)	Low (Multiple operators)
Francis et al., 2021 [48]	Low (RCT)	Very low (Attrition 0.0%)	Moderate (One blinded operator)
White, 1996 [49]	Low (RCT)	High (Attrition 20%)	Moderate (One blinded operator)
Ho and Niederman, 1997 [50]	Low (RCT)	Very low (Attrition 0.0%)	Moderate (One blinded operator)
Heasman et al., 1998 [51]	Low (RCT)	Very low (Attrition 0.0%)	Low (Multiple operators)
Pucher et al., 1999 [52]	Low (RCT)	Moderate (Attrition 13%)	Moderate (One blinded operator)
Borutta et al., 2002 [53]	Low (RCT)	Low (Attrition 5%)	Moderate (One blinded operator)
Hickman et al., 2002 [54]	Low (RCT)	Low (Attrition 5%)	Moderate (One blinded operator)
Costa et al., 2007 [55]	Low (RCT)	Very low (Attrition 0.0%)	Moderate (One blinded operator)
Silvestrini Biavati et al., 2010 [56]	Low (RCT)	Very low (Attrition 0.0%)	Low (Multiple operators)
Marini et al., 2014 [57]	Low (RCT)	Very low (Attrition 0.0%)	Moderate (One blinded operator)
Zingler et al., 2014 [58]	Low (RCT)	Low (Attrition 8%)	Moderate (One blinded operator)
Mylonopoulou et al., 2021 [59]	Low (RCT)	Very low (Attrition 0.0%)	Moderate (One blinded operator)

## Data Availability

The data presented in this study are publicly available from PubMed. All of the study data are indexed and were accessed by the study authors online.
